# Analysis of 30 patients with persistent or recurrent squamous cell carcinoma of the cervix within one year after concurrent chemoradiotherapy

**DOI:** 10.7497/j.issn.2095-3941.2013.04.007

**Published:** 2013-12

**Authors:** Shi-Ping Liu, Jia-Xin Yang, Dong-Yan Cao, Keng Shen

**Affiliations:** Department of Obstetrics and Gynecology, Peking Union Medical College Hospital, Peking Union College, Chinese Academy of Medical Science, Beijing 100730, China

**Keywords:** Carcinoma, squamous cell, cervix uteri, chemoradiotherapy, neoplasm recurrence, local, risk factors, prognosis

## Abstract

**Objective:**

To investigate the recurrence sites, risk factors, and prognosis of patients with persistent or recurrent squamous cell carcinoma (SCC) of the cervix within one year after undergoing concurrent chemoradiotherapy (CCRT).

**Methods:**

Clinical data of 30 patients with persistent or recurrent SCC of the cervix within one year after CCRT between July 2006 and July 2011 were analyzed retrospectively. These data were compared with those of 35 SCC cases with no signs of recurrence after complete remission. These 35 patients were treated during the same period (between 2006 and 2011) and selected randomly.

**Results:**

Among these 30 patients, 25 exhibited distant metastases of which 14 were observed within 6 months after CCRT. Univariate analysis showed higher incidence of pelvic or para-aortic lymphadenectasis and SCC-ag >10 ng/mL in the group with persistent or recurrent disease before treatment (*P*<0.01). Multivariate analysis by logistic regression revealed that the pre-therapeutic pelvic or para-aortic lymph node enlargement and SCC-ag >10 ng/mL were the independent risk factors. Palliative chemotherapy was the main treatment option for patients with persistent or recurrent disease. The 2-year survival rate was 21.7%, and the median survival time was 17 months.

**Conclusion:**

Patients with persistent or recurrent SCC of the cervix after CCRT exhibited a high rate of distant metastasis with poor prognosis. The pre-therapeutic pelvic or para-aortic lymph node enlargement and SCC-ag >10 ng/mL were identified as the independent risk factors for persistent or recurrent SCC within 1 year after CCRT.

## Introduction

Concurrent chemoradiotherapy (CCRT) is the main and effective treatment means for advanced cervical cancer. However, there are still a number of patients developing persistent or recurrent disease after definitive CCRT. Once this occurs, the prognosis is very poor. This paper retrospectively analyzed the recurrence sites, risk factors and prognosis of 30 patients with persistent or recurrent squamous cell carcinoma (SCC) of the cervix within 1 year after CCRT, aiming at providing some guidance for the clinical practice.

## Materials and methods

### Clinical data

A total of 30 patients with persistent or recurrent SCC of the cervix within 1 year after undergoing the initial chemoradiotherapy at Peking Union Medical College Hospital were assigned to the recurrence group (age range: 29-66 years; median age: 45.5 years). These patients underwent chemoradiotherapy between July 2006 and July 2011 and complete clinical data were obtained from that period. A persistent lesion, or a new lesion which occurred within 3 months after the radiotherapy, was taken as the index for persistent disease, and the new tumor occurring after complete remission accompanied by progressively elevated serum SCCAg levels was taken as the recurrence index. A total of 35 SCC patients were randomly selected from those who were concurrently treated in the same hospital and showed no signs of recurrence, as confirmed by physical examination, imaging, and serum SCCAg levels after more than 1-year follow-up. These 35 patients were assigned to the control group (age range: 34-66 years; median age: 47 years).

### Methods

Clinical characteristics including age, FIGO stage, tumor size, lymph node status, serum SCCAg level before radiotherapy, radiotherapy time, radiotherapy dose, and cycle of concurrent chemotherapy were analyzed. Pre-radiotherapy lymph node status was assessed according to the imaging results (abdominal and pelvic CT, MRI or PET-CT). Pelvic and/or para-aortic lymphadenectasis detected by the imaging examination were deemed as lymph node metastasis. Serum SCCAg level of all patients was detected within 2 weeks before the start of the radiotherapy. The tumor differentiation level was not included in the statistical analysis since some pathology reports failed to provide it. Upon the end of radiotherapy, the follow-up was started. The following time points marked the end of follow-up: July 31, 2012, when patients died, or loss to follow-up.

### Statistical analysis

All data were processed with SPSS 19.0, and statistical analyses of categorical variables were performed using Chi-square test or Fisher exact test. Logistic regression was adopted for multivariate analysis, and statistical significance was defined as *P*<0.05. Kaplan-Meier method was employed for survival analysis.

## Results

### Radiotherapy

All of the patients were treated with pelvic radiation and vaginal brachytherapy. For patients with suspected para-aortic lymph node metastasis, extended-field pelvic and para-aortic radiotherapy was performed. The radiotherapy durations of 4 patients from each group were longer than 56 d, and the average total point A dose was 89.4 and 87.5 Gy, respectively. The differences in both the radiotherapy duration and dose were not statistically significant (*P*>0.05). During the radiotherapy, all patients received concurrent cisplatin chemotherapy (40 mg/m^2^, once a week). The average cycle of concurrent chemotherapy for each group was 4.4 and 4.7, respectively, with no significant difference (*P*>0.05).

### Recurrence sites

Among the 30 patients in the recurrence group, distant metastasis occurred in 25 patients (83.3%), and recurrence sites of 5 patients (16.7%) were confined to the pelvic cavity. Of the 25 patients with distant metastasis, 21 patients (70.0%) developed only distant metastasis without pelvic failure or recurrence (**Table 1**). Notably, 14 patients suffered from distant metastasis within 6 months after undergoing radiotherapy.

**Table 1:** Sites of recurrence, *n* (%)

Recurrence pattern	*n* (%)
Local failure or recurrence	5 (16.7)
Limited to cervix	2 (6.7)
Beyond cervix but inside pelvis	3 (10.0)
Distant metastasis	21 (70.0)
Limited to PALN	1 (3.3)
Limited to SCLN (with or without SCLN metastasis)	3 (10.0)
Beyond SCLN and PALN (lung, bone, liver, ect.)	17 (56.7)
Synchronous local and distant relapse	4 (13.3)

PALN, para-aortic lymph node; SCLN, supra-clavicular lymph node.

### Risk factors

Univariate analysis demonstrated that the proportion of the patients with pre-therapeutic pelvic and/or para-aortic lymphadenectasis and SCCAg >10 ng/mL was higher in the recurrence group than in the control group (*P*<0.05, **Table 2**). Multivariate analysis by logistic regression also showed that the pre-chemoradiotherapy lymphadenectasis and SCCAg >10 ng/mL were the independent risk factors for persistent or recurrent SCC of the cervix within 1 year after radiotherapy.

**Table 2:** Univariate analysis of potential risk factors for patients with persistent or recurrent disease, *n* (%)

Clinical data	Recurrence group (*n*=30)	Control group (*n*=35)	*χ*^2^	*P*
Age (years)			0.032	0.857
≤35	3 (10.0)	2 (5.7)		
>35	27 (90.0)	33 (94.3)		
Stage			2.681	0.262
IB	3 (10.0)	1 (2.9)		
II	18 (60.0)	27 (77.1)		
III	9 (30.0)	7 (20.0)		
Tumor size (cm)			0.707	0.401
≤4	21 (70.0)	21 (60.0)		
>4	9 (30.0)	14 (40.0)		
Lymph node			19.726	<0.001
Positive	20 (66.7)	5 (16.7)		
Negative	10 (33.3)	30 (83.3)		
SCCAg (ng/mL)			7.05	0.005
≤10	16 (53.3)	7 (20.0)		
>10	14 (46.6)	28 (80.0)		
Radiation time (d)			0	1
≤56	26 (86.7)	31 (88.6)		
>56	4 (13.3)	4 (11.4)		
Radiation dose (Gy)			─	0.495
≥86	30 (100)	33 (94.3)		
<86	0 (0)	2 (5.7)		
Cycles of chemotherapy			0.372	0.542
≥4	24 (80.0)	31 (88.6)		
<4	6 (20.0)	4 (11.4)		

### Prognosis

**Figure 1:**
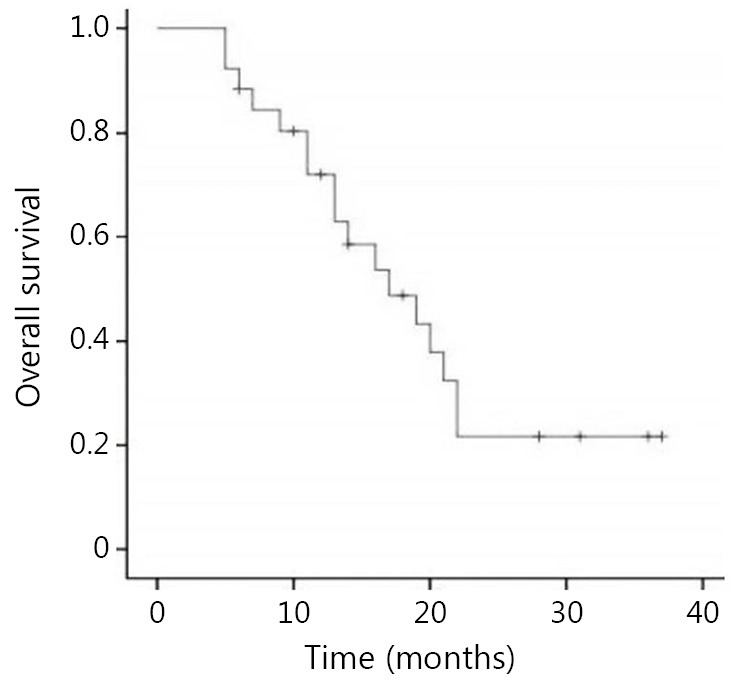
Overall survival of patients with persistent or recurrent disease.

In the recurrence group, 4 patients were lost to follow-up. Among the remaining 26 patients, only 1 patient with local recurrence limited to the cervix was completely relieved from the disease after 6 courses of chemotherapy, and showed no signs of recurrence during the 31-month follow-up. Two patients received 12 and 13 interrupted chemotherapy courses, respectively, during which the disease was partially relieved or stabilized, and the follow-up for both patients lasted for 3 years. Meanwhile, 19 patients suffered from disease progression or could not stand the chemotherapy after undergoing an average of 3.3 cycles of chemotherapy, and were thus given symptomatic and supportive treatment. Two patients received symptomatic and supportive treatment after palliative radiotherapy, and 2 patients received simply the symptomatic and supportive treatment due to poor general conditions. The 2-year survival rate of the patients with persistent or recurrent disease was 21.7%, with median survival period of 17 months (**Figure 1**).

## Discussion

Since CCR was clinically used, the survival rate of patients with advanced cervical cancer has greatly increased in recent years. However, many patients still suffer from persistent or recurrent disease after CCR. Eifel *et al.*^1^ reported that the local and distant recurrence rate of locally advanced cervical cancer after CCR was 17% and 18%, respectively. The prognosis of such patients was very poor. Hong *et al.*^2^ reported that the 5-year survival rate of patients with distant metastasis or local recurrence after radiotherapy was about 10%. Furthermore, the shorter the interval between the end of the therapy and the recurrence was, the poorer the prognosis was^3^^-^^5^. In the current study, the 2-year survival rate of the patients with persistent or recurrent SCC of the cervix within 1 year after CCR was 21.7%, and the median survival period of these patients was 17 months.

In recent years, some scholars have studied the risk factors for radiotherapy failure of cervical cancer. Hirakawa *et al.*^6^ summarized 108 patients with SCC of the cervix, and found that elevated SCCAg at the end of the radiotherapy, pre-radiotherapy anemia and lymph node metastasis were associated with distant metastasis. They also identified lymph node metastasis as the risk factor for intrapelvic recurrence. Hong *et al.*^2^ reported that young age and late-stage disease were associated with local recurrence, while late-stage disease, SCCAg >10 ng/mL, and pelvic lymph node metastasis were the independent risk factors for distant metastasis. Zhang *et al.*^7^ conducted a retrospective analysis of 213 patients, and found that tumor diameter ≥4 cm and tumor pathological grade 3 were the risk factors for local failure or recurrence after radiotherapy. Yu *et al.*^8^ reported that late FIGO stage, tumor size, and cycle of concurrent chemotherapy influenced local control of advanced cervical cancer. Song *et al.*^9^ reported that the radiotherapy time >56 days was detrimental to pelvic control in cervical cancer patients undergoing CCR; however, the lengthening of the radiotherapy time did not increase the distant metastasis rate of the patients. Huang *et al.*^10^ reported that the pre-treatment serum carcinoembryonic antigen (CEA) ≥10 ng/mL was the risk factor for para-aortic lymph node recurrence following definitive CCR for SCC of the uterine cervix. Kang *et al.*^11^, meanwhile, established a model for predicting the distant metastasis risk of locally advanced cervical cancer after CCR (pelvic and para-aortic nodal positivity on FDG-PET, non-squamous cell histology, and pretreatment serum SCC antigen levels). This model was of high resolution and calibration level. In addition, several reports^12^ showed that the mean apparent diffusion coefficient value on diffusion weighted magnetic resonance imaging can be used to identify patients with a risk of disease recurrence. To the best of our knowledge, however, there have been no reports specially describing the risk factors for persistent or recurrent cervical carcinomas within a short period after CCR. According to the preliminary results of the current study, pre-radiotherapy pelvic and/or para-aortic lymphadenectasis and SCCAg >10 ng/mL were the risk factors for persistent or recurrent SCC of the cervix within a short period after radiotherapy. This result is consistent with the results of the above-mentioned studies to some extent, since over 80% of the recurrences occurred within 2 years after radiotherapy. However, our data showed that the distant metastasis occurred earlier and indicated a high probability. Among the 21 patients with only distant metastasis, 14 patients developed distant metastasis within 6 months after radiotherapy, which was much shorter than the distant metastasis occurrence time reported by other studies^2^ (the median time was about 18 months). Given this, the possibility that these patients might have micrometastasis before radiotherapy could not be excluded. Treatment options for patients with persistent or recurrent disease are various, including chemotherapy, radiotherapy, surgery and palliative treatment and the specific conditions of the patients play a key role in the treatment choice. In this study, the specific conditions of our patients, such as high distant metastasis rate (83.3%), persistent disease or recurrence within a short period after radiotherapy, and so on, indicated that the following treatment consisted mainly of systematic chemotherapy. At present, the cisplatin-based chemotherapy is used as the standard treatment option for recurrent cervical cancer, but its effect is not good enough^13^^-^^16^. One study^17^ showed that the supportive care-based therapy was potentially more cost-effective than the current standard doublet chemotherapy for patients with recurrent cervical cancer.

In order to improve the prognosis of such patients, sufficient and timely primary CCR is warranted. Besides, the evaluation and treatment strategies should be further improved. As described above, patients who developed distant metastasis within a short period after radiotherapy may suffer from neglected metastasis. Thus, it is necessary to undertake a comprehensive assessment of the patients with such high risk factors as pelvic and/or para-aortic lymph node metastasis and markedly elevated SCCAg before radiotherapy, in order to discover the micrometastasis and develop a rational treatment plan. Some reports^13^^,^^18^^-^^20^ showed that chemotherapy (including neoadjuvant chemotherapy and consolidation chemotherapy) can improve the prognosis of the cervical carcinoma patient affected by unfavorable prognosis factors. Hong *et al.*^2^ reported that the prognosis of the patients with recurrent disease limited to para-aortic lymph node was better than that of patients with supra-clavicular lymph node metastasis. Furthermore, they reported that the prognosis of patients with recurrent disease restrained to the cervix was better than that of patients with recurrent disease beyond the cervix. Thus, intense follow-up of patients with a high risk for recurrence is required in order to discover earlier recurrence and make timely remedy measures.
